# Synergistic broad-spectrum antibacterial activity of *Hypoxis hemerocallidea-*derived silver nanoparticles and streptomycin against respiratory pathobionts

**DOI:** 10.1038/s41598-021-93978-z

**Published:** 2021-07-27

**Authors:** Oluwole S. Aremu, T. Qwebani-Ogunleye, Lebogang Katata-Seru, Zimbili Mkhize, John F. Trant

**Affiliations:** 1 Institute of Traditional Knowledge and Traditional Medicine, Vaal University of Technology Science and Technology Park, 5 Moshoeshoe Road, Sebokeng, 1911 South Africa; 2 Department of Chemistry, North-West University, Mafikeng, South Africa; 3 Department of Chemistry and Biochemistry, University of Windsor, 401 Sunset Avenue, Windsor, ON N9B 3P4 Canada

**Keywords:** Antimicrobials, Nanoparticles, Infection

## Abstract

Respiratory tract infections arise due to the introduction of microbes into the airway, disrupting the normal, healthy, complex interdependent microbiome. The selective disruption of this community can be either beneficial or dangerous. Nanoparticles are a potential tool for modifying this population. Coated silver nanoparticles (AgNPs) were synthesized using ethanolic extracts of *Hypoxis hemerocallidea* (EEHH), a Southern African plant used extensively in traditional medicine and the source of many bioactive secondary metabolites. The room temperature reaction between silver nitrate and EEHH forms largely spherical AgNPs with an average diameter of 6–20 nm. These nanoparticles show similar levels of antibacterial activity as the broad-spectrum antibiotic streptomycin against *Bacillus cereus*, *Streptococcus pneumonia*, *Escherichia coli*, *Pseudomonas aeuroginosa*, and *Moraxella catarrhalis.* However, the AgNPs synergistically increase the antibacterial activity of streptomycin when they are applied in combination (30–52%). AgNPs are reiterated to be promising dual-function antibiotics, synergistically enhancing activity while also acting as delivery agents for small molecules.

## Introduction

Acute respiratory infections and lung disease remain a deadly public health issue with an estimated 3.5 million deaths in 2019 prior to the emergence of SARS-CoV-2^1^. The death rates peak during both infancy and late adulthood. Over 2 million pediatric cases have been reported, more than for any other public health disease^2,3^. Lung disease and acute middle ear infections also harm global health. Many pediatric and adult patients experience inflammation from middle ear infection with reoccurrence, which is common in developing countries^4^.

Pathobionts, a mixed population of bacteria universally present in the human upper respiratory tract, include *Streptococcus pneumoniae* (*pneumococcus*), *Hemophilus influenza*, *Moraxella catarrhalis*, and *Staphylococcus aureus*^5,6^. Changes in this respiratory microbial community can open niches allowing for colonization by new pathogens that can lead to respiratory disease, especially in individuals with compromised or naïve immune systems^7^. The use of metallic nanoparticles in personal protective equipment and/or consumer goods could prove useful in limiting the availability of these pathogens in the environment, protecting these populations from infection.

Nanoparticle synthesis from inorganic salts requires the addition of reducing and capping β-agents to provide the organic passivating shell around the metal core. Although many reagents can be used, employing complex matrices from plant extracts offers several advantages including likely biocompatibility, ready access to the starting material from non petroleum sources, and, often, low cost by repurposing otherwise waste material^8^. Some of the phytochemicals can survive the synthetic process endowing the nanoparticles with additional functionality beyond that provided by the metal itself^9,10^. Fortunately, many secondary metabolites from plants, like carbohydrates and flavonoids, have been shown to be capable of reducing of Ag^+^ to AgNPs^11^. Furthermore, the stability of the resulting AgNPs and the kinetics of their growth and consequent resulting size, can be tuned through the changing the identity of the capping agents. The capping agents determine these parameters through their electrostatic and non-covalent bonding interactions with the nascent AgNP surface^12,13^.

**Figure 1:**
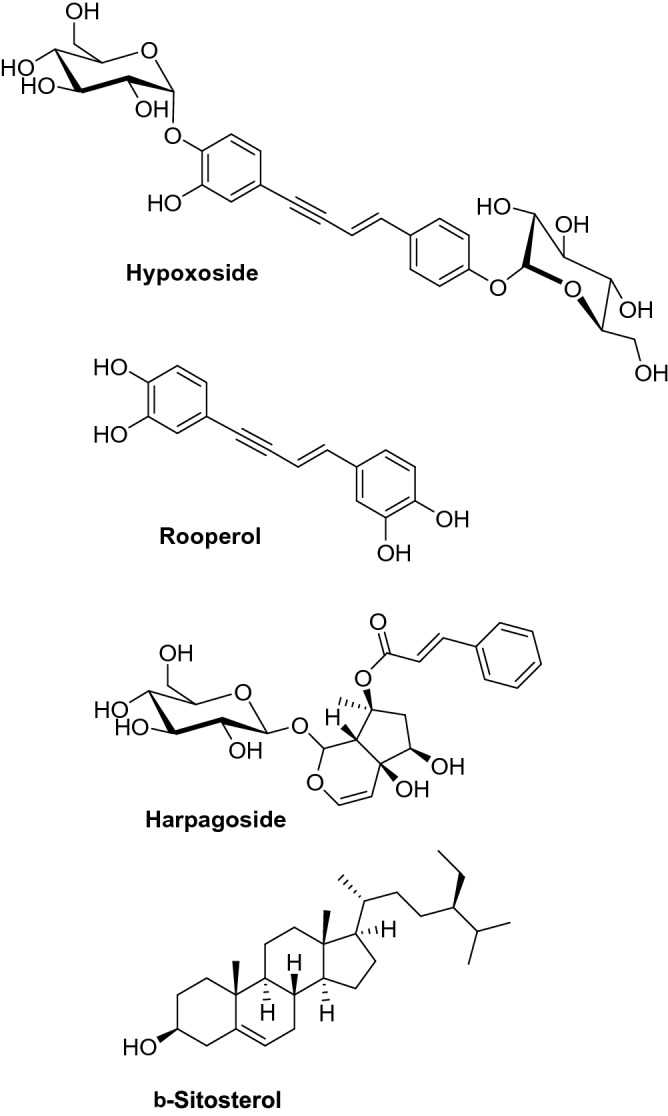
Structures of major known secondary metabolites in *Hypoxis hemerocallidea.*

*Hypoxis hemerocallidea* (HH), the “African potato,” is used in traditional medicine across Southern Africa, especially for endocrine gland dysplasia^14^, and as a purgative and remedy for delirium, bad dreams, impotency, and apprehension by the Zulu^15,16^. HH is a vascular plant with bright yellow flowers inspiring its common name: "yellow stars." HH contains some rather unusual phytochemicals like the ene-yne-containing hypoxoside and rooperol, the iridoid glycoside harpagoside, and the unusual steroid β-sitosterol (Fig. 1), which collectively have demonstrated pharmacological potential for antibacterial, anti-inflammatory, antioxidant, and anticancer activity^17–19^. It is often consumed by HIV/AIDS patients to boost their immunity and improve their general wellbeing^20^; however, as its constituents have been shown to inhibit Cytochorme p450 metabolism, it could possibly interfere with many retroviral drugs^21^, although the evidence remains inconclusive to date^22^. Best practice recommends that its use be discussed with clinicians when starting antiretroviral therapy^23^. This species could be used in the synthesis of AgNPs to manufacture a synergistic product with therapeutic potential*.* To the best of our knowledge, AgNPs have never been prepared using HH, and the synergistic impact of co-administering HH ethanolic extract (EEHH) and its AgNPs with antibiotics to treat pathogens has never been studied. This is the goal of this work.

## Materials and methods

### Extraction of phytochemicals from *Hypoxis heamerocallidea* for the synthesis of AgNPs

Fresh HH corms, cultivated, were purchased from local commercial farms in Sebokeng in Gauteng, South Africa. Cultivation of these plants was conducted using normal commercial farming practices in line with South African regulations. Authentication of their identity was conducted by botanist Dr. Bukola Aremu of the School of Biological Sciences, North-West University, Mafikeng, South Africa, and the University of Windsor, Windsor, Canada. The corms were then extensively washed under running water, dried at room temperature (24 °C), and pulverized into a powder with a commercial blender. Pulverized corms (500 g) were soaked in absolute ethanol (1.5 L) with mechanical shaking for 72 h. The extract was filtered to remove the powder, then concentrated under reduced pressure using a rotary evaporator at 70.1 °C to obtain a dark brown powder. After drying under high vacuum for an additional 16 h to constant mass, 23.6 g of a dark brown powder was obtained^18^. This fraction, the ethanolic extract of *Hypoxis heamerocallidea* is referred to as EEHH.

### Synthesis of AgNPs-HH

A stock solution of EEHH was prepared by suspending 2 g of the crude powder in 50 mL of 70% ethanol (v/v). Next, it was sonicated for 10 min to solubilize the solution to a translucent deep yellow solution with no observable turbidity. To a 40 mL aliquot of this solution was then added 400 µL of 0.1 M AgNO_3_ in one portion with vigorous stirring. The reaction mixture immediately changed to a dark brown colour, indicative of the formation of the AgNPs. The process was carried out at ambient room temperature open to the atmosphere, and progress was monitored by measuring the UV–Vis spectrum of the reactant mixture at hourly intervals over 4 h according to our previously reported procedure^24^. With no further changes being observed between 3 and 4 h, the particles were purified by dialysis (against water, cut-off of 3.5 kDa), then dried by lyophilization and stored until resuspended when needed.

### Phytochemical screening

A qualitative phytochemical analysis of the EEHH (independent of the presence of the silver) was carried out using standard procedures^25–27^. The crude extract was screened for the presence of saponins, tannins, phenols, coumarins, flavonoids, terpenoids, glycosides, alkaloids, and proteins. To test for tannins, we performed a ferric chloride test by placing 5 mL of the extracts from nine plants inside test tubes. A few drops of 0.1% ferric chloride were added. The presence of brownish green or blue–black color indicated the presence of tannins in the sample. For sterols and triterpenoids, we performed the Salkowski test. 5 mL of the extracts from nine plants were placed in test tubes, and 2 mL of chloroform and 3 mL of concentrated sulfuric acid were added consecutively. We shook them well and allowed them to stand for a few minutes. Red color in the lower layer indicates the presence of sterols, and yellow color in the lower layer indicates the presence of triterpenoids. To test for flavonoids, we performed an alkaline reagent test. A few drops of 1% liquor ammonia were put in a test tube containing the sample. The emergence of a yellow color confirmed the presence of flavonoids. To test for glycosides, we performed a bromine water test. We added 5 mL of bromine water to the test extract solutions, and a yellow precipitate indicated the presence of glycosides. To test for saponins, we did a foam test. To 10 mL of the sample, 3 mL of distilled water was added and shaken well to obtain a froth. A few drops of olive oil were added to the froth, and the formation of an emulsion indicates the presence of saponins. To test for cardiac glycosides, we did the Kellar–Kiliani test. To 5 mL of a sample, 2 mL of glacial acetic acid containing a drop of ferric chloride was added. This was followed by the addition of 1 mL of concentrated sulfuric acid. A brown ring obtained indicates a positive result for the test. To test for alkaloids, 0.1 mg of the extract was added to 6 mL of dilute hydrochloric acid (1 M) and boiled, cooled, and filtered. The filtrate was divided into three parts and subjected to the following tests. To the first aliquot, 2 drops of Dragendorff’s reagent were added. The formation of red precipitate indicated the presence of alkaloids. To the second aliquot, 2 drops of Meyer's reagent were added. A creamy white precipitate revealed the presence of alkaloids. To the third aliquot, 2 drops of Wagner's reagent were added. The formation of a reddish-brown precipitate indicated the presence of alkaloids. To test for phenol, 0.5 mL of extract was added to 5 mL of Foalin Ciocletu reagent and 4 mL of aqueous sodium carbonate. The generation of a blue colour indicated the presence of phenol.

### Characterization of the nanoparticles

The absorption spectra of the AgNPs were measured between 300 and 700 nm using a PerkinElmer (Germany) 365 UV–Vis spectrometer at 24 °C. The morphology of the AgNPs was examined on a JEOL 3010 high-resolution transmission electron microscope equipped with energy-dispersive X-ray (EDX) functionality (Bruker, Germany). The FTIR spectra of the crude extract and the AgNPs were obtained on a Bruker Alpha-P FTIR spectrophotometer (Germany) from 500 to 4000 cm^−1^. The structural characterization of the AgNPs was carried out using an X-ray diffractometer. XRD analysis was conducted using Bruker equipment with monochromatic Cu kα radiation (λ = 1:5406 Å) at 40 kV. Scanning was conducted in the region of 20–100 2θ angles. Dynamic light scattering (Malvern Zetasizer Nano-ZS) was used to analyze the zeta potential of the synthesized AgNPs. For the DLS measurements, powder AgNPs were resuspended in distilled water and sonicated for 15–20 min to properly disperse the particles in water. Zeta potential, and hydrodynamic diameter values were obtained from the triplicate analysis of the nanoparticles in the aqueous media^24^. Gas Chromatography–Mass Spectrometry (GC–MS) analysis was performed using a Bruker GC–TOF–MS Gas Chromatography coupled with a 5973 Mass selective detector. The capillary column (Rxi-5SilMS) with an internal diameter of 30 × 25 mm and 0.2 μm film thickness (Restek, Bellefonte, PA, USA) was used. Ultrahigh purity helium (Afrox, South Africa) was used as the carrier gas at a constant flow rate of 1.0 mL/min and a linear velocity of 37 cm/s. An aliquot of 1 μL of sample diluted in the respective solvents was injected into the column with an inlet temperature of 250 °C in a splitless mode at time of 30 s. An initial oven temperature of 40 °C was set and programmed to increase up to 300 °C at the rate of 10 °C per min with a holding time of 3 min at each increment. The electron ionization mode of 70 eV (EI+) and electron multiplier/detector voltage at 1750 V was used to operate the mass spectrometry. The other operating parameters were as follows: ion source temperature of 230 °C, MS transfer line temperature 280 °C, MS solvent delay time of 5 min and mass acquisition range of 40–550 DA and data acquisition rate of 10 spectra/S. The compounds were identified by direct comparison of the mass spectrum of the analyte at a particular retention time to that of reference standards found in the National Institute of Standards and Technology (NIST) library. The area percentage of each component was calculated by comparing its average peak area to the total areas obtained^28^.

### Antibacterial susceptibility

The antibacterial activity of the EEHH, AgNPs, and a combination treatment of AgNPs and streptomycin (50 µL:50 µL), were investigated against five pathogenic bacterial strains: Gram-positive *S. pneumoniae* (ATCC 27336) and *Bacillus cereus* (ATCC 10876); and Gram-negative *M. catarrhalis* (ATCC 25240), *Escherichia coli* (ATCC 25922), and *Pseudomonas aeruginosa* (ATCC 27853)^29^. Using the disc diffusion technique, pure cultures of these microorganisms were sub-cultured on nutrient agar and incubated at 37 °C for 24 h.

Fresh overnight cultures were inoculated on Mueller Hinton agar (MHA) plates using sterile swabs and allowed to stand for 20 min. Wells of 6-mm diameter were made on MHA plates with the bacterial lawn. Each well was filled with 50 μL of different concentrations (50, 100, and 150 μg/mL) EEHH in distilled water and HH AgNPs in dimethyl sulfoxide (DMSO) prepared from 10 mg/mL stock. DMSO (5%) was used as the negative control, and streptomycin (10 μg/mL) served as the reference standard. The plates were incubated at 37 °C for 24 h, and the diameters of the inhibition zones around the wells were measured. Experiments were carried out in triplicate to reduce error^30^. The minimum inhibition concentration (MIC) and minimum bactericidal concentration (MBC) of green synthesized AgNPs were determined using the modified method described in the CLSI guideline (2012)^31^. The MIC test was performed in a 96-well round bottom microtiter plate using standard broth microdilution methods while the MBC test was performed on the MHA plates. The bacterial inoculums were adjusted to the concentration of 10^6^ CFU/mL. For the MIC test, 100 μL of the synthesized AgNPs stock solution (500 μg/mL) was added and diluted twofold with the bacterial inoculums in 100 μL of MHB started from column 12 to column 3. Column 12 of the microtiter plate contained the highest concentration of AgNPs, while column 3 contained the lowest concentration. Column 1 served as negative control (only medium) and the column 2 served as positive control (medium and bacterial inoculums). Each well of the microtiter plate was added with 30 μL of the resazurin solution and incubated at 37 °C for 24 h. Any colour changes were observed. Blue/purple colour indicated no bacterial growth while pink/colourless indicated bacterial growth. The MIC value was taken at the lowest concentration of antibacterial agents that inhibits the growth of bacteria (colour remained in blue).

Streptomycin (Sigma, St. Louis) treatments were all conducted at 10 μg/mL. This was prepared as needed from a stock solution by diluting tenfold with the assay media. The stock solution was prepared by transferring 10 mg of streptomycin into a 1 mL volumetric flask, and making up the volume with a 1:1 ethanol: water solution, providing a 10 mg/mL solution. This was then diluted 100-fold by transferring 10 μL of this solution to a new 1 mL volumetric flask, and making up the volume with the 50% ethanolic solution. This provided the 100 μg/mL stock.

The MBC was defined as the lowest concentration of the antibacterial agents that completely kill the bacteria. MBC test was performed by plating the suspension from each well of microtiter plates that exhibited no colour change into MHA plate. The plates were incubated at 37 °C for 24 h. The lowest concentration with no visible growths on the MHA plate was taken as MBC value. The log of reduction RF_value_ were enumerated accordingly.

## Results and discussion

### Phytochemical screening

The results from the qualitative analysis of the EEHH are provided as Table 1. The EEHH ethanolic extract was positive for alkaloids, flavonoids, steroids, phenols, terpenes, glycosides, carbohydrates, saponins, and tannins as expected, but the tests were negative for cardiac glycosides^32^.

**Table 1:** Phytochemical analysis of the crude extract of *Hypoxis hemerocallidea.*

Metabolite	Ethanolic extract
Glycosides	+
Flavonoids	+
Alkaloids	+
Terpenes	+
Steroids	+
Tannins	+
Saponins	+
Phenol	+
Cardiac glycosides	−

### GC–MS profiling

The volatile phytochemicals present in the ethanolic crude extract were subjected to GC–MS analysis (Table 2). The extract is dominated with high molecular mass unsaturated fatty acid palmitoleic acid, unusual sugar d-allose, and the unusual chlorinated long-chain hydrocarbon 2-chloroethyl linoleate. Considerable amounts of siloxanes were observed, these are likely environmental contaminants from the growing conditions, but are not naturally produced by the plant.

**Table 2:** Major constituents of the chemical composition of the ethanolic crude extract of HH corms using GC–MS.

No	Name	Retention time (m)	% Area
1	Palmitoleic acid	19.31	12.84
2	d-Allose	12.56	10.58
3	2-Chloroethyl linoleate	19.250	10.16
4	Pentadecanoic acid	15.59	4.83
5	1,2,3,5-Cyclohexanetetrol	14.01	3.61
6	O-geranyl-β-d-Mannofuranoside	21.68	2.68
7	5-Hydroxymethylfurfural	8.79	2.50
8	(*E,E,E*)*-*9-Octadecenoic acid, 1,2,3-propanetriyl ester	23.84	2.47
9	Stearic acid	19.49	2.18
10	5,6,6-trimethyl-Undeca-3,4-diene-2,10-dione	13.40	2.11
11	3-Deoxy-d-mannoic lactone	13.83	1.91
12	Benzaldehyde, 3,4-dihydroxy-	13.54	1.61
13	Octadecamethyl-cyclononasiloxane	23.09	1.44
14	2,5-Monomethylene-l-rhamnitol	10.03	1.41
15	Hexadecamethylheptasiloxane	24.05	1.41
16	eicosamethyl-Cyclodecasiloxane	22.07	1.26
17	N-Nitrosoazacyclononane	6.63	1.25
18	Hexadecamethylheptasiloxane	20.99	1.19
19	Tetracosamethyl-cyclododecasiloxane	24.93	1.19

Other trace components < 1.00%.

### UV–Vis analysis

**Figure 2:**
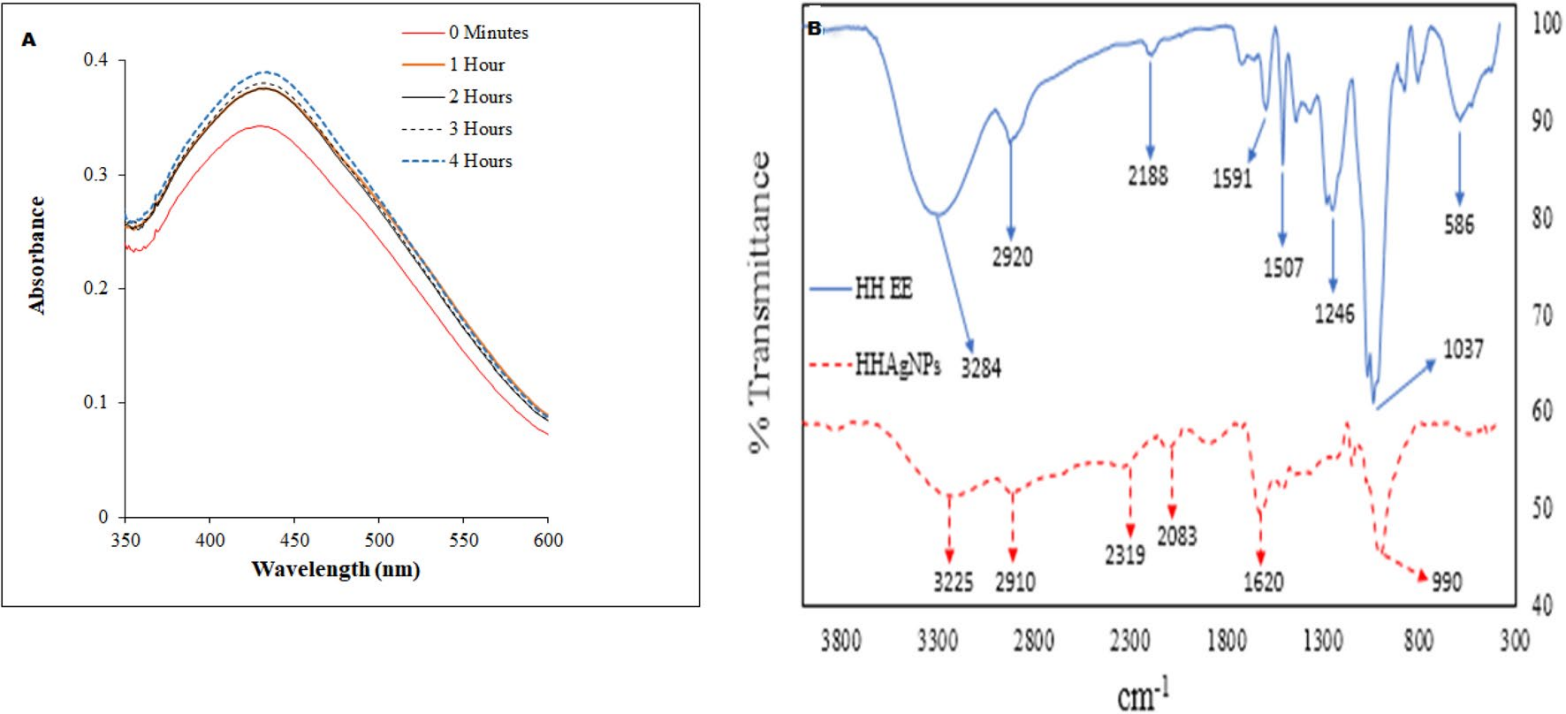
Nanoparticle formation: (**A**) UV spectrum of the AgNPs formed, (**B**) surface charge: FTIR spectra of the extract and AgNPs.

The UV–vis absorption spectra of the AgNPs as a function of time during synthesis are provided as Fig. 2A. EEHH-AgNPs produced surface plasmon resonance (SPR) peaks at 430–434 nm, which is consistent with no agglomeration. The generated AgNPs were stable over the reaction period, and the UV–vis peak stabilized at 434 nm without further movement beyond 4 h indicating the presence of a steady state. However, because the response time increased, the SPR peak position became bathochromically shifted, indicating a gradual increase in nanoparticle size^32–34^.

### FTIR analysis

The FTIR spectrum is consistent with the incorporation of the secondary metabolites and their degradation products into the AgNPs as the same functional groups are present in both the extract and the nanoparticles. As noted by others, many classes of phytochemicals can reduce silver salts to metallic silver, while others can act as capping agents. This will, of course, affect the chemical structure of these organic compounds, and these transitions are noted by the changes in the relative intensity of the vibrational bands.

The pronounced peaks of the extract were at 3284, 2920, 1591, 1507, 1246, 1037, and 586 cm^−1^, whereas those of the AgNPs were at 3225, 2910, 1620, and 990 cm^−1^. The broad vibration at 3284/3225 cm^−1^ is typical of hydroxyl groups on carbohydrates, flavonoids, and saponins. The peaks at 2920/2910 cm^−1^ arise from aliphatic C–H stretches in alkyl groups, 1591 and 1507 cm^−1^ from aromatic ortho disubstituted C–H stretches, 1246 cm^-1^ is typical of phenol O–H stretches, while 1037 cm^−1^ suggests the presence of aliphatic ethers and alcohol C–O bonds^35,36^. These alcohols and phenols are probably involved in the reduction of ionic silver to zero-valent AgNPs and decrease upon reaction (Fig. 2B)^37^.

The reduction of the relative intensity of the aliphatic C–H stretching region during AgNPs synthesis suggests conversion of C–H bonds in the phytocompounds to multiple bonds or oxidation^38^. The FTIR analysis is consistent with the formation of AgNPs through reduction of Ag(I) to Ag (0) with concomitant oxidation of the HH phytochemicals.

Phytochemical screening of crude EEHH confirmed the presence of flavonoids and carbohydrates. Flavonoids release free reactive hydrogen during their tautomeric transformations (keto-enol rearrangement), which can assist in the reduction of AgNPs^39^. Furthermore, alcohols facilitate the reduction of ionic silver to zero-valent AgNPs as they oxidize to carbonyls^40^.

### TEM, SAED, and EDX analysis

**Figure 3:**
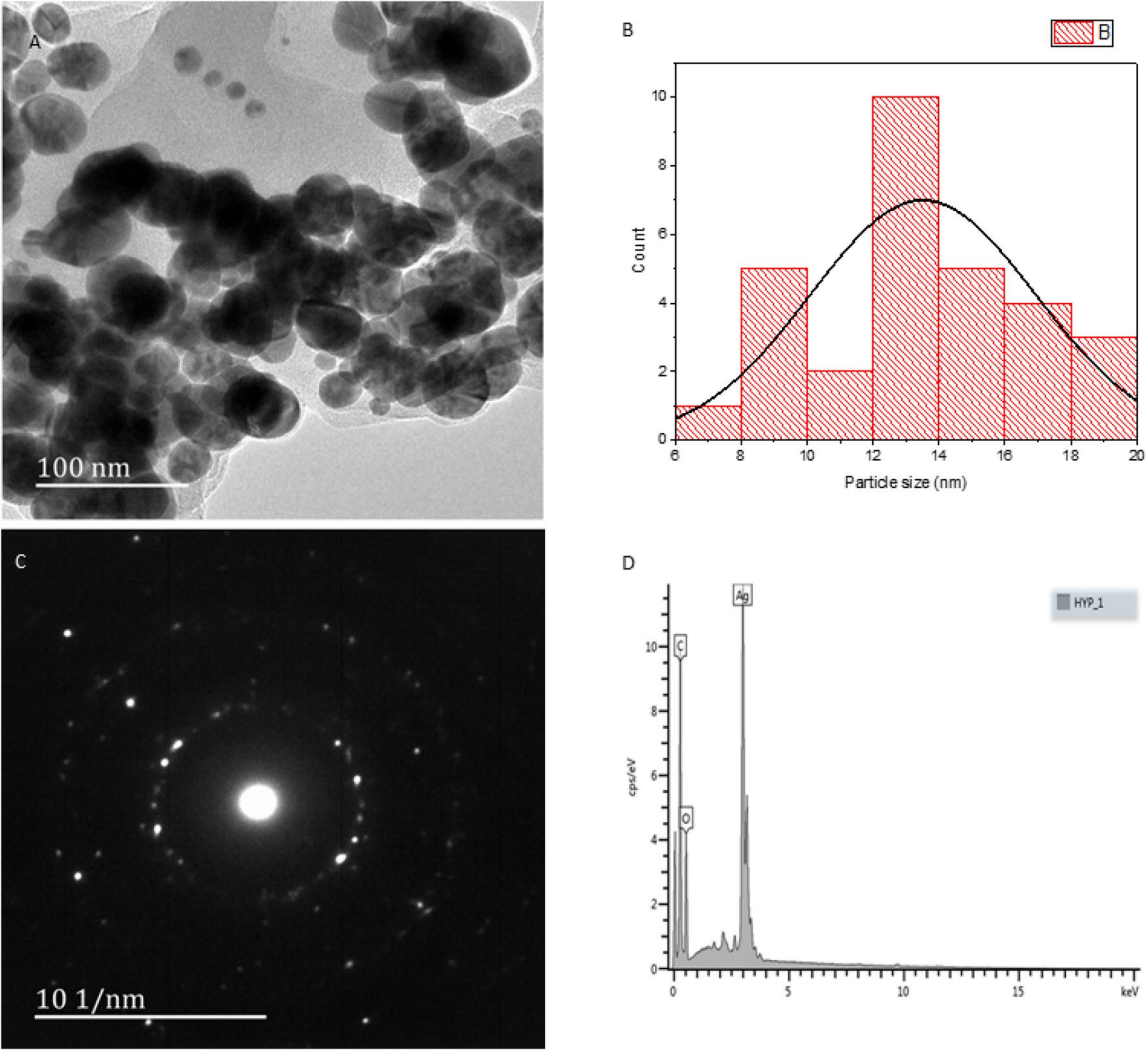
Morphology: (**A**) TEM, (**B**) Particle size distribution (**C**) TEM-SAED, (**D**) EDX.

TEM analysis supports a largely spherical morphology for the AgNPs (Fig. 3A). Through sizing 30 randomly selected particles observed by TEM we observed the mode was between 12 and 14 nm, with the mean value being 13.3 nm (Fig. 3B). As noted above, these are core–shell structures; this is suggested in the TEM images, but the distinct rings found in the SAED patterns (Fig. 3C) of the AgNPs confirm the polycrystalline property of the as-synthesized AgNPs^41^. Elemental composition analysis by EDX supports the contention that the nanoparticles comprise a metallic core with an organic shell: strong silver (Ag) signals and weaker signals from C and O atoms are consistent with this hypothesis. There is no significant contamination from the nitrogen in the nitrate or from other opportunistic metals. The position of the signal at 13cps/eV suggest that the silver core is crystalline rather than amorphous (Fig. 3D)^41^.

### X-ray diffraction

**Figure 4:**
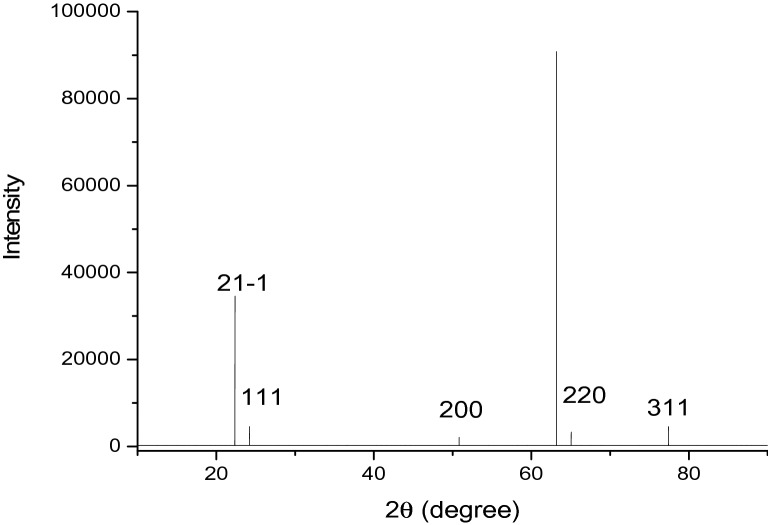
XRD pattern of green synthesized AgNPs using *HH.*

The XRD pattern of the AgNPs shows sharp diffraction peaks corresponding to the (111), (200), (220), and (311) crystal planes (Fig. 4), which are associated with the face-centered cubic lattice of silver. The XRD profile indicates that our materials crystallize in a monoclinic phase, and this spectrum is in line with those reported for other organic-silver nanoparticles prepared from other botanical extracts^42–44^. This confirms the formation of silver nanocrystals as the metallic core of the nanoparticles^24^.

### Dynamic light scattering and Zeta potential analysis of the nanoparticles

**Figure 5:**
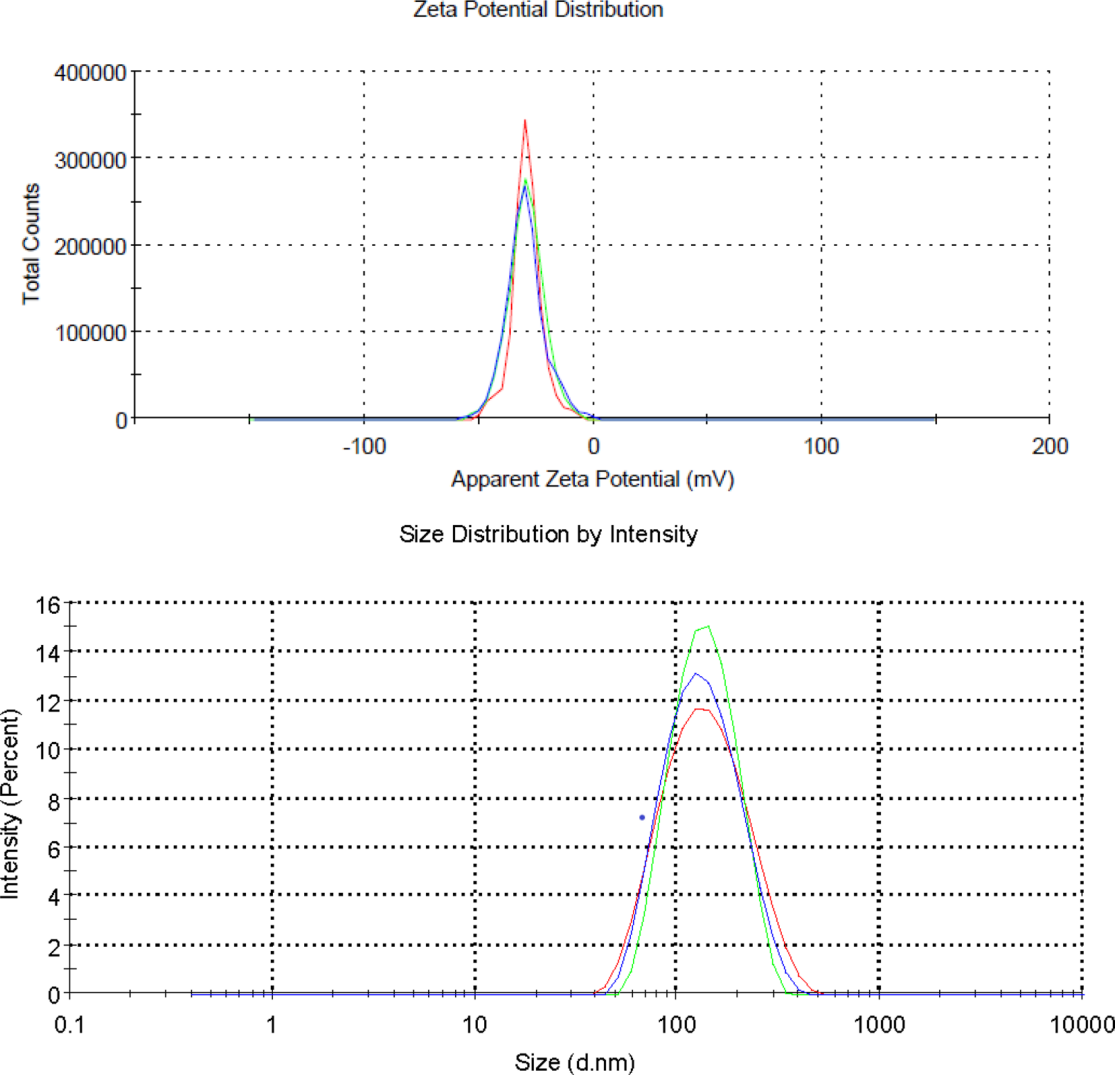
Zeta potential and size distribution of the AgNPs. Analyses were conducted in triplicate for three freshly prepared samples, and each run is plotted in the different colours.

The zeta potential value of *HH* mediated AgNPs in aqueous suspension was established as − 29.2 mV (Fig. 5). This suggests that the surface of the nanoparticles is negatively charged and that the particles are uniformly dispersed in the aqueous medium. The high negative value is evident of the extreme stability of the nanoparticles because of electrostatic repulsive forces between the particles. Zeta potential value of about − 29 mV ensures a high energy barrier for the stabilization of the nanosuspension. DLS suggests a hydrodynamic diameter of 119 nm with a polydispersity index of 0.188. This is an order of magnitude larger than that observed by TEM. Although there are many small particles generated, the TEM images suggest the presence of larger structures. These might move as an assembly in solution together to provide this larger observed size in solution.

### Antibacterial susceptibility

**Figure 6:**
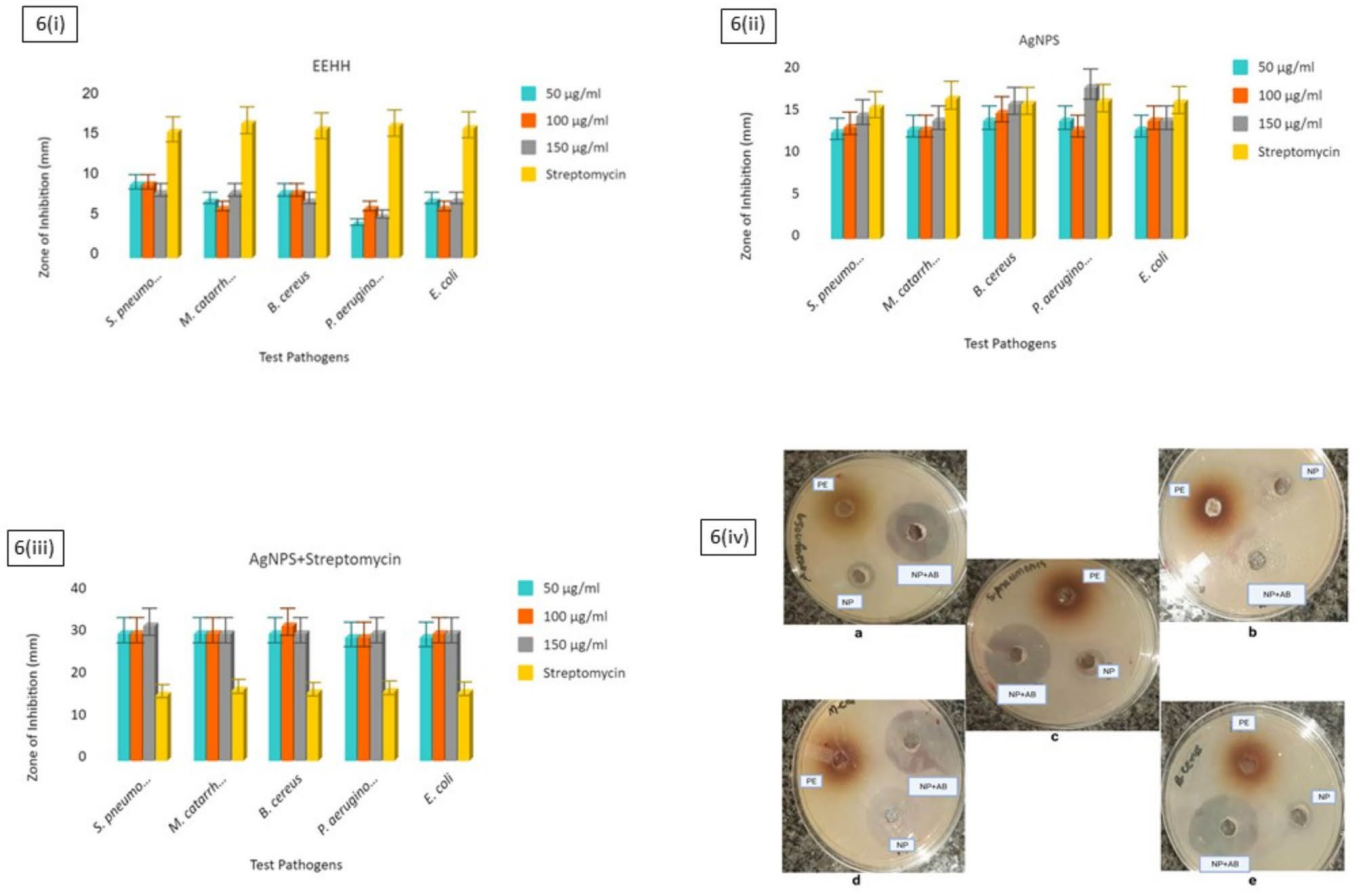
The antibacterial activity of (**i**) EEHH, (**ii**) AgNPs, (**iii**) AgNPs and Streptomycin (10 μg/mL for all experiments), (**iv**) zone of inhibition on agar plate.

The antibacterial activity of the crude extracts, AgNPs, and the AgNPs co-administered with streptomycin, were investigated against both Gram-positive and Gram-negative bacteria. This was quantified using a standard Kirby–Bauer disc diffusion assay with DMSO as the negative control and pure streptomycin as the positive control (Fig. 6).

The extract alone only shows mild activity. The components of EEHH have been a traditional medicine, and like most secondary plant metabolites likely have some role in plant defense against infection. However, the AgNPs show good activity against all bacteria regardless of Gram-status and are similar in potency to streptomycin. However, when streptomycin and the AgNPs are used together, the effect is synergistic: the area of disinfection is considerably greater than a simple addition of their individual activities would indicate. DMSO showed no activity with no area of inhibition (data not shown) confirming the viability of the tested bacteria strains. Silver salts have long been known to be potent antimicrobials, and the development of silver nanoparticles, with their far higher surface area of activated silver metal, has greatly accelerated their investigation^45^. However, the overuse of silver can decrease its efficacy against microorganisms as they develop resistance^46^.

The MIC result (Table 3) shows the bacteriostatic effect of the AgNP at 0.156 µg/mL for *Streptococcus pneumonia*, *Moraxella catarrhalis* and *Pseudomonas aeruginosa* and 0.312 µg/mL upon exposure to *Escherichia coli* and *Bacillus cereus*. The bactericidal effect of the AgNP ranges from 0.312 to 5 µg/mL for *Streptococcus pneumonia*, *Moraxella catarrhalis* and *Pseudomonas aeruginosa* and 0.625–5 µg/mL with *Escherichia coli* and *Bacillus cereus*. The log of reduction RF_value_ > 4 in all the bacteria challenge irrespective of their Gram status. The nanoparticle thus possessed high efficacy with a percentage greater than 90%.

**Table 3:** Bacterial activity of the AgNP (µg/mL) with the mean log of reduction (RF).

Bacteria	MIC (µg/mL)	MBC (µg/mL)	RF_value_
*Streptococcus pneumonia*	0.156	0.312	5.36E+00 (100%)
*Bacillus cereus*	0.312	0.625	5.06E+00 (95%)
*Moraxella catarrhalis*	0.156	0.312	4.89E+00 (91%)
*Escherichia coli*	0.312	0.625	5.36E+00 (100%)
*Pseudomonas aeruginosa*	0.156	0.312	5.19E+00 (97%)

This synergistic effect is expected and has been seen with other silver nanoparticles^47,48^. The mechanism of action of the AgNPs is through adsorption to the bacterial cell membranes, followed by passive penetration into the bacteria. The surface then becomes transiently exposed, and they can cause damage by interacting strongly with essential phosphorous and sulfur-containing compounds such as DNA and proteins, resulting in bacterial cell death^49^. They could also assist in preventing the bacteria from initiating gene expression changes due to the presence of the streptomycin, or streptomycin might better enter the cell by adsorbing to the surface of the nanoparticle and being carried into the cell itself along with the toxic silver particle^50^.

## Conclusions

HH corm extract is readily obtained in large amounts from even small amounts of plant material (5% mass recovery from crude material) and can be used to initiate the solution-phase synthesis of low dispersity AgNPs under ambient conditions.

These biosynthesized AgNPs showed no agglomeration and had sizes typically ranging from 6 to 20 nm with a roughly spherical or ovoid shape. These HH-AgNPs show broad spectrum antibacterial activity against common respiratory pathobionts and synergistically enhance the antibacterial activity of streptomycin. We propose that these could be useful agents for transporting largely insoluble antibiotics in the body as potential biologically-active drug delivery vehicles, but much more analysis needs to be conducted.
